# Human Umbilical Cord–Mesenchymal Stem Cells Combined With Low Dosage Nintedanib Rather Than Using Alone Mitigates Pulmonary Fibrosis in Mice

**DOI:** 10.1155/sci/9445735

**Published:** 2025-01-07

**Authors:** Huijun Qiu, Rong Zhang, Daozhu Si, Yi Shu, Jiang Liu, Yunqiu Xia, Ou Zhou, Wen Tan, Ke Yang, Daiyin Tian, Zhengxiu Luo, Enmei Liu, Lin Zou, Zhou Fu, Danyi Peng

**Affiliations:** ^1^Department of Respiratory Medicine Children's Hospital of Chongqing Medical University, National Clinical Research Center for Child Health and Disorders, Ministry of Education Key Laboratory of Child Development and Disorders, Chongqing 400014, China; ^2^Centre for Clinical Molecular Medicine, Children's Hospital of Chongqing Medical University, Chongqing 400014, China; ^3^Chongqing Engineering Research Centre of Stem Cell Therapy, Chongqing 400014, China

**Keywords:** cell proliferation, human umbilical cord–mesenchymal stem cells (hUC–MSCs), nintedanib, pulmonary fibrosis

## Abstract

Pulmonary fibrosis (PF) is a lethal pathological change of fibrotic interstitial lung diseases (ILDs) with abundant fibroblasts proliferation after severely or continually alveolar epithelial cells (AECs) injury. Barely therapies are helpful for PF. Here we use bleomycin intratracheally injection to model PF with or without human umbilical cord–mesenchymal stem cells (hUC–MSCs) and/or nintedanib intervention. RNA-Seq followed with real-time PCR and western blot were used to find out the specific possible mechanisms of the effects of hUC–MSC and nintedanib on PF. Immunostaining, cell counting kit-8 (CCK-8), and 5-bromo-2′-deoxyuridine (BrdU) incorporation assay were used to detect the cell proliferation in vivo or in vitro separately. We found that hUC–MSCs alone had prophylactic, but not therapeutic effects on bleomycin induced mouse PF. Nevertheless, the combination therapy of hUC–MSCs and low-dose nintedanib significantly improved survival and reversed lung fibrosis in PF model mice. The factors secreted by hUC–MSCs have promotional effects on the proliferation both of fibroblasts and AECs. Nintedanib could hamper the facilitation of fibroblasts caused by hUC–MSCs without influence on AECs proliferation, which might be related with the inhibition on FGFR, PDGFR, and VEGFR activities. Our study indicated that the combination therapy of hUC–MSCs and nintedanib should be a promising strategy for PF.

## 1. Introduction

Interstitial lung diseases (ILD) are a group of pulmonary conditions that affect the alveolar structures, pulmonary interstitium, and/or small airways. Multiple factors, such as environmental and occupational exposures, infections, drugs, radiation, and genetic predisposition, have been implicated in the pathogenesis of ILD [1]. Pulmonary fibrosis (PF) is the mutual pathological change of fibrotic ILD [2]. Most patients with PF show a progressive decline in pulmonary function, which eventually leads to respiratory failure and death [3]. The incidence of ILD and PF increased significantly in recent years, but until now the treatment options of PF have been limited.

Conventional therapy consisting of glucocorticoids or immunosuppressive drugs has been affirmed ineffective. Currently, lung transplantation is considered to be an effective treatment approach, but only in limited number of patients, with a 5 year posttransplantation survival rate of about 50%. Recently, two novel disease-modifying therapies, nintedanib and pirfenidone, have shown promising results [2, 4]. Nonetheless, no significant effect on overall mortality was seen. Besides, the concerns based on current costs and adverse effects may limit feasibility and use of the two drugs [5]. Further explorations of the PF therapies are exigent.

Mesenchymal stromal cells (MSCs) are adult multipotent cells which can be isolated from bone marrow, adipose tissue, umbilical cord (UC), dental pulp, and other tissues [6]. Over the past decade, MSCs have been exploited as therapeutic vectors to treat a wide variety of diseases, especially the autoimmune rheumatic diseases [7, 8] and fibrotic disorders [9]. Notwithstanding the immunomodulatory properties [6], the effects of MSCs on PF have still been controversial [10–13]. The protective role of MSCs in rodent bleomycin PF models is affirmative, which offer better support for the application of MSCs in acute inflammatory phase rather than the chronic fibrotic phase of lung injury [10, 11]. While administration of MSCs had no effect on established fibrotic changes [12]. Further, they could acquire a myofibroblast phenotype [13].

As MSCs may be a double-edged sword in the treatment of lung fibrosis, combining it with other drug or bioengineering tech to treat diseases is a better choice compared with using MSCs alone. The combining therapies of MSCs and bioengineering scaffolds are mostly applied to the regeneration and repair of skin wounds, spinal cord or peripheral nerve injuries, and bone defects [14]. Otherwise, there is less research on the combination therapies of MSCs and chemicals. But human UC (hUC)–MSCs have been used in combination with ursodeoxycholic acid in a clinical trial for the patients with primary biliary cholangitis [15]. The preliminary data related to safety and efficacy of the trail herald the prospects of the combination therapy of MSCs and chemicals.

In this study, we observed that administration of hUC–MSCs alone at 7 days after modeling with bleomycin was ineffective. Then we combined hUC–MSCs and low dosage nintedanib and found that the combination could reverse the established lung fibrosis in mice, which might be related with the influence on proliferation of type II alveolar epithelial cells (AECIIs).

## 2. Materials and Methods

### 2.1. Treatment of Animal Models

**Figure 1:**
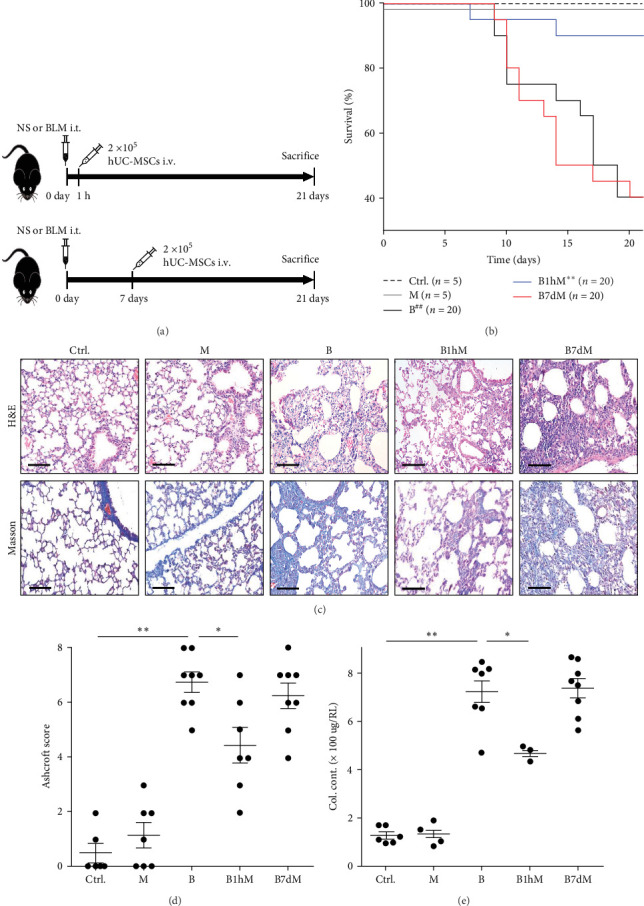
Human umbilical cord–mesenchymal stem cells (hUC–MSCs) alone has prophylactic, but not therapeutic effect on pulmonary fibrosis (PF) induced by bleomycin in mice. (A) Schematic of hUC–MSCs administration at different times after bleomycin modeling. NS, saline. BLM, bleomycin (3.0 mg/kg). i.t., injected intratracheally. i.v., injected intravenously. 2 × 10^5^ hUC–MSCs in 80 μl NS had been delivered per mice for specific groups. (B) Kaplan–Meier survival curves of the mouse with different administration. Dotted line “Ctrl.”: NS i.t. alone. Gray line “M”: hUC–MSCs i.v. alone. Black line “B”: bleomycin i.t. alone. Blue line “B1hM”: hUC–MSCs i.v. at 1 h after bleomycin modeling. Red line “B7dM”: hUC–MSCs i.v. at 7 days after bleomycin modeling. (^##^*p* < 0.01 compared with Ctrl. group, *⁣*^*∗∗*^*p* < 0.01 compared with B Group) (C) Hematoxylin–eosin (H&E) and Masson's trichrome staining of lung sections from the indicated mouse groups. Scale bars: 50 µm. (D) Ashcroft score of the pathological staining. (E) Collagen contents (Col. Cont.) in the right lungs (RLs) assessed by Sircol assay. Shown is a representative of at least three independent experiments with *n* = 6 mice for “Ctrl.” group, *n* = 7 mice for “M” group and *n* = 5 mice for “B1hM” group, while *n* = 8 mice/group for “B”, and “B7dM” groups. For D and E, shown is mean ± SEM. *⁣*^*∗*^*p* < 0.05, *⁣*^*∗∗*^*p* < 0.01.

The C57BL/6J mouse included in the experiments were at 8–10 weeks. They were maintained on normal water ad libitum. A total of 3.0 mg/kg of bleomycin (Sigma, USA) was injected intratracheally (i.t.) using an intubation procedure as previously described [16]. Control animals received the same volume of saline. For observing the effects of hUC–MSCs interference, the hUC–MSCs (2 × 10^5^) or saline (80 μl) were injected intravenously (i.v.) at 1 h or 7 days after modeling (Figure 1A). For observing the effects of nintedanib administration, the nintedanib (10 or 30 mg/kg) was daily orally deserved for 2 weeks from the 7th day of modeling with or without hUC–MSCs. All mouse experiments were carried out under specific pathogen-free conditions at Children's Hospital of Chongqing Medical University in a facility approved by the China Association for the Accreditation of Laboratory Animal Care. All the protocols were performed in accordance with the recommendations in the Guide for the Care and Use of Laboratory Animals (Library of Congress Control Number: 2010940400, revised 2011) guidelines and relevant regulations.

### 2.2. Histopathologic Examination and Immunostaining

Lungs were fixed with 10% buffered formalin, dehydrated and paraffin embedded, and 4 μm sections were collected on microscope slides. Hematoxylin–eosin (H&E) and Masson's trichrome stains were used for analysis of pathologic changes. The severity of fibrosis was evaluated in stained sections by an individual who was blinded to the management and genotypes of the mice. To evaluate the extent of fibrotic changes, a quantitative fibrotic scale (Ashcroft scale) was used [17]. Immunofluorescence (IFC) on paraffin-embedded lung tissue sections were performed with primary antibodies as previously described [18]. Antibodies were used as following: anti-*α*-SMA (1:100), anti-SPC (1:100), and anti-Ki67 (1:200). Antibodies were all purchased from Abcam.

### 2.3. Sircol Collagen Assay

Total lung collagen levels were determined by measuring soluble collagen in each right lung using a Sircol collagen assay kit (Biocolor, Country Antrim, UK) according to the manufacturer's instructions.

### 2.4. RNA-Seq, Different Gene Analysis, and Gene Ontology (GO) Analysis

Total RNA was extracted from the samples by Trizol reagent (Invitrogen) separately. The RNA quality was checked by Agilent 2200 and kept at −80°C. The cDNA libraries were constructed for each RNA sample using the TruSeq Stranded mRNA Library Prep Kit (Illumina, Inc.) according to the manufacturer's instructions. The libraries were quality controlled with Agilent 2200 and sequenced by HiSeq X (Illumina, San Diego, CA) on a 150 bp paired-end run. Clean reads were obtained from the raw reads by removing the adaptor sequences and low-quality reads. HTseq [19] was used to get gene counts and RPKM method was used to determine the gene expression. We applied EBSeq algorithm [20] to filter the differentially expressed genes, after the significant analysis, *p*-value and FDR analysis were subjected to the following criteria: (i) fold change > 1.5 or <0.667 and (ii) FDR < 0.05. GO analysis was performed to facilitate elucidating the biological implications of the differentially expressed genes in the experiment [21]. We downloaded the GO annotations from GO (http://www.geneontology.org/). Fisher's exact test was applied to identify the significant GO categories (*p*-value < 0.05).

### 2.5. RT-PCR

Total RNA was isolated using the RNAiso Plus kit (Takara, Japan), and cDNA was synthesized by reverse transcription with SuperScript II as described by the manufacturer (Takara, Japan). Quantitative real-time RT-PCR was performed according to the manufacturer's protocol (QuantiFast SYBR Green PCR Kit, Qiagen, German). Primers were shown in *Supporting Information 1*: Table S1. The relative expression of a defined gene was calculated using the *ΔΔ*CT method.

### 2.6. Cell Line Culture

Human lung fibroblast cell line HFL1 and AECII line HPAEpiC was purchased from the Wuhan SAIOS Biotechnology Co., Ltd. They were cultured in ordinary culture medium or the hUC–MSCs conditional medium (CM) with or without different concentrations of nintedanib (50 or 100 nM) for 96 h. For cell proliferation detecting, cell counting kit-8 (CCK-8; Dojindo, Kumamoto, Japan) or 5-bromo-2′-deoxyuridine (BrdU; Roche, Germany) incorporation assay were performed as described previously [16]. Different treatment groups of cells were also collected for the immunoblot analysis.

### 2.7. Immunoblot Analysis

Lysates of particular cells were analyzed by western blotting using the following antibodies: anti-FGFR2 (1:1000, proteintech, China), anti-PDGFR*α* (1:1000, proteintech, China), anti-PDGFR*β* (1:1000, proteintech, China), anti-VEGFR (1:1000, CST, USA), anti-phospho FGFR2 (Ser782; 1:1000, Invitrogen, USA), anti-phospho PDGFR*α* Tyr849/ PDGFR*β* Tyr857 (1:1000, CST, USA), anti-phospho VEGFR (1:1000, CST, USA), and anti-*β*-actin (1:1000, CST, USA). To quantify protein expression, densitometry was performed on the lanes using Gene Tools software (Syngene, Cambridge, UK).

### 2.8. Statistical Analysis

All values are expressed as means ± SEM. Multiple comparisons of parametric data were performed using one-way ANOVA at specific time-points and two-way at different time-points, followed by Bonferroni multiple test between groups. Statistical differences were analyzed using SPSS software (version 16). *p* values of less than 0.05 was considered to be statistically significant. Kaplane–Meier survival analysis was performed using Prism 5 (GraphPad Software, La Jolla, CA) and statistical significance was determined by log-rank test.

## 3. Results

### 3.1. hUC–MSC Alone has Prophylactic but Not Therapeutic Effect on PF Induced by Bleomycin in Mice

hUC–MSCs had been intratracheal instillation at 1 h or 7 days after modeling in different groups (Figure 1A). Mouse modeled by PBS and treated with hUC–MSCs at 7 days after modeling were used to confirm the safety of hUC–MSCS treatment. The results showed that hUC–MSCs administration at 7 days after PF modeling had no effect on the median survival time of PF mouse, while early access of hUC–MSCs could improve it (Figure 1B). The pathological damages and collagen deposition caused by bleomycin could not be reversed by hUC–MSCs using at 7 days after modeling. But early hUC–MSCs application could limit the progression of PF caused by bleomycin obviously (Figure 1C–E). Which meant that hUC–MSCs alone had prophylactic but not therapeutic effects on mouse PF induced by bleomycin. But the hUC–MSCs are nearly not available for the patients at the early stage of PF. So we continued to figure out the possible reasons for the ineffectiveness of single hUC–MSCs treatment on late stage of PF.

**Figure 2:**
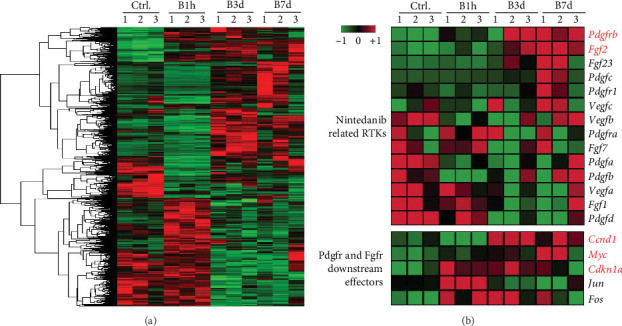
Platelet-derived growth factor receptor-*β* (Pdgfrb) and fibroblast growth factor 2 (Fgf2) were obviously higher in the 7 days after modeling group than that of the 1 h after modeling group. RNA-Seq had been applied for the differentially expressed genes detection in lung tissues at various times after bleomycin modeling. (A) Hotmap of the differentially expressed genes in lung tissue at the four indicated times after bleomycin modeling. (B) Hotmap of the main RTKs (Fgfr, Pdgfr, and Vegfr), their ligands and the downstream effectors of Pdgfr and Fgfr. Ctrl., lung tissue obtained at 7 days after intratracheally injected saline alone. B1h, B3d, and B7d are lung tissue obtained at 1 h, 3 days, or 7 days after bleomycin modeling separately. *n* = 3 mice each group.

Whole pulmonary expression profiles were compared between the mice of normal saline control group, 1 h after modeling group, 3 days after modeling group, and 7 days after modeling group. GO analysis of the genes with increase or decrease expression trends among these four groups showed that many of these genes were participated in the pathways in cancer. Looking into the pathways, we caught sight of two familiar genes, platelet-derived growth factor receptor-*β* (Pdgfrb) and fibroblast growth factor 2 (Fgf2), which were the target genes of nintedanib. Nintedanib is an intracellular inhibitor that targets multiple tyrosine kinases, including the vascular endothelial growth factor (VEGF), FGF, and PDGF receptors. It had been approved for the treatment of idiopathic PF (IPF) by US Food and Drug Administration recently. Our data showed that the expression of Pdgfrb and Fgf2 in the whole lung increased gradually as the time after modeling, which were obviously higher in the 7 days after modeling group than that of the control group and 1 h after modeling group (Figure 2B). The downstream effectors of Pdgfr and Fgfr, including cyclin D1 (Ccnd1), Myc proto-oncogene protein (Myc) and cyclin-dependent kinase inhibitor 1A (Cdkn1a), were also involved in the differentially expressed gene list among the four groups, which had been reported to affect cell proliferation. And the restriction of fibroblasts proliferation was one of the therapeutic effects of nintedanib. Although the guideline put a high value on the potential benefit of nintedanib on the rate of FVC decline, no significant effect on overall mortality was seen. The feasibility of this treatment was also limited by the high cost and commonly reported adverse effects, specifically diarrhea, as well [5]. Therefore, we used the combination of nintedanib and hUC–MSCs to try to reduce the dosage of nintedanib and enhance the therapeutic effect.

### 3.2. The Combination of Low-Dose Nintedanib and hUC–MSCs had Therapeutic Effect on PF in Mice Caused by Bleomycin

**Figure 3:**
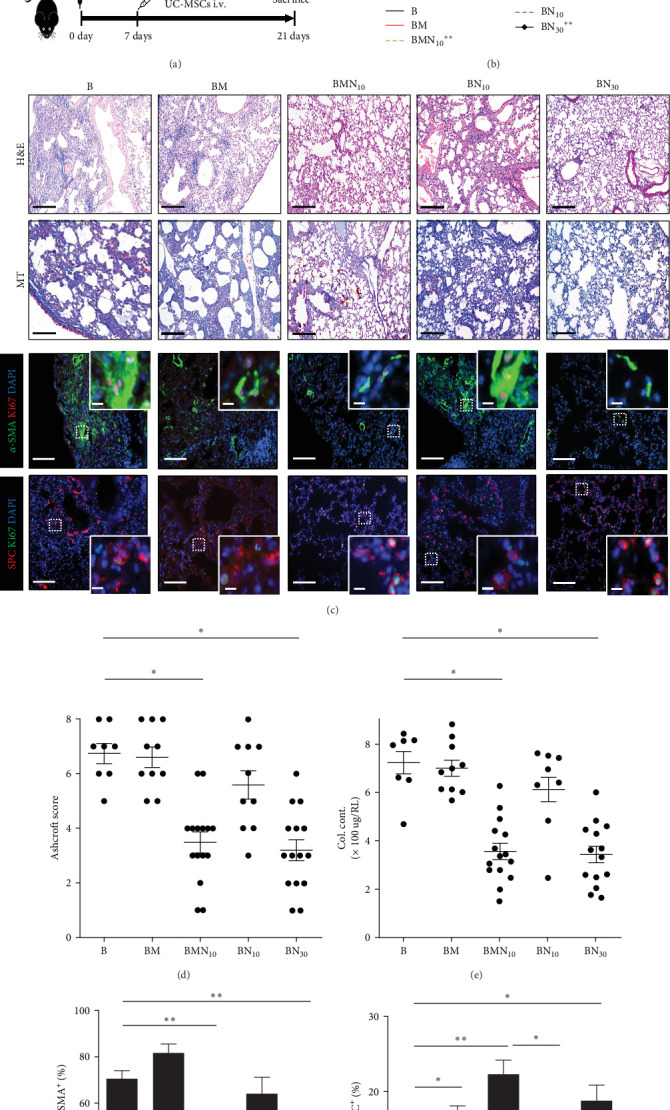
The combination of human umbilical cord–mesenchymal stem cells (hUC–MSCs) and low-dose nintedanib has therapeutic effect on pulmonary fibrosis (PF) induced by bleomycin in mice. (A) Schematic of nintedanib and hUC–MSCs combination treatment on mice lung fibrosis induced by bleomycin. For model control group “B”, 3.0 mg/kg bleomycin had been injected intratracheally (i.t.). For group treated by hUC–MSCs alone “BM,” 2 × 10^5^ hUC–MSCs in 80 μl saline had been injected intravenously (i.v.) at 7 days after modeling. For combination treatment group “BMN_10_,” 2 × 10^5^ hUC–MSCs in 80 μl saline had been i.v. at 7 days after modeling and 10 mg/kg nintedanib had been delivered intragastically (i.g.) daily from the 7 to 20 days after modeling. For the group treated by nintedanib alone “BN_10_ and BN_30_,” 10 or 30 mg/kg nintedanib had been delivered i.g. daily from the 7 to 20 days after modeling without hUC–MSCs. (B) Kaplan–Meier survival curves of the mouse from indicated groups. (*⁣*^*∗∗*^*p* < 0.01 compared with model control group “B”). (C) Representative images of Hematoxylin–eosin (H&E) and Masson's trichrome staining and immunostaining of lung sections from the indicated mouse groups. Scale bars: 50 µm. Except for the enlarged view scale bars: 250 µm. (D) Ashcroft score of the pathological staining. (E) Collagen contents (Col. Cont.) in the right lungs (RLs) assessed by Sircol assay. (F) The percentage of Ki67 positive cells among *α*-SMA positive cell. (G) The percentage of Ki67 positive cells among SPC positive cell. Shown is a representative of at least three independent experiments with *n* = 20 mice/group. For D to G, shown is mean ± SEM. *⁣*^*∗*^*p* < 0.05, *⁣*^*∗∗*^*p* < 0.01.

The median survival time of PF mouse had be prolong with 30 mg/kg nintedanib intragastric administration daily from the 7th day after modeling. Moreover, the pulmonary pathological changes and acid-soluble collagen deposition caused by bleomycin were also alleviated by 30 mg/kg nintedanib. Interestingly, we found that although low-dose nintedanib (10 mg/kg) had no therapeutic effect on PF, the combination administration of it and hUC–MSCs could reduce the Ashcroft score and collagen deposition in PF mouse, which was comparable to that of 30 mg/kg nintedanib (Figure 3A–E). Moreover, we had seen more AECII cells and less myofibroblasts proliferation in the lungs of combination therapeutic group than that of low-dose nintedanib group (Figure 3C,F,G).

### 3.3. hUC–MSCs had Positive Effect on Proliferation of Both AECIIs and Fibroblasts

**Figure 4:**
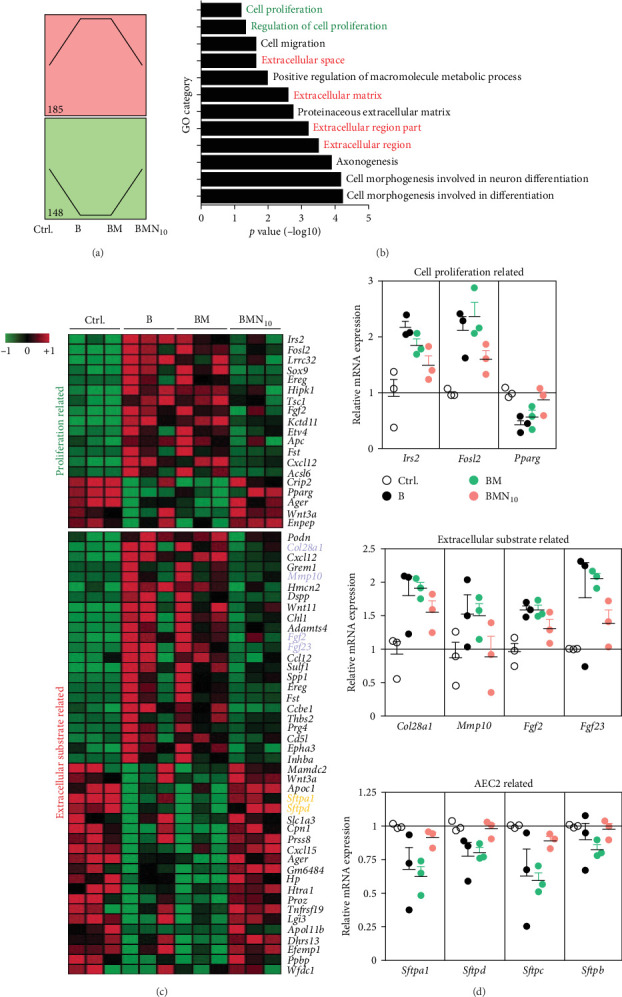
The combination therapy downregulated some cell proliferation and extracellular substrate related genes, while upregulated markers of alveolar epithelial cells (AECs). RNA-Seq had been applied for the differentially expressed genes detection in lung tissues from indicated mouse groups. (A) Tow trends that genes used for subsequent analysis matched. Trend 1 (185 genes), the expression of which was higher in the modeling group “B” than normal control group “Ctrl.,” but had no difference between group B and hUC–MSCs treated alone group “BM,” while decreased in the combination therapy group “BMN_10_.” Trend 2 (148 genes), the expression of which was lower in “B” group than “Ctrl.” group, but had no difference between “B” group and “BM” group, while increased in “BMN_10_” group. (B) Gene Ontology (GO) analysis of the genes. (C) Hotmap of cell proliferation related genes and extracellular substrate related genes. (D) RT-PCR were used to verify the mRNA expression of representative genes related to cell proliferation and extracellular substrate and the markers of AECs. Shown is mean ± SEM. Shown is a representative of at least three independent experiments with *n* = 3 mice each group.

To find out the reasons why the combination therapy rather than single low-dose nintedanib had therapeutic effects on PF caused by bleomycin in mice, whole pulmonary expression profiles were compared between the mice of normal saline control group, modeling group, PF mouse treated by hUC–MSCs alone group, and PF mouse treated with combination therapy group. The genes which could match one of the expression trends below were picked out to made GO analysis. Trend 1, the expression of which was higher in the modeling group than normal control group, but had no difference between modeling group and hUC–MSCs treated alone group, while decreased in the combination therapy group. Trend 2, the expression of which was lower in the modeling group than normal control group, but had no difference between modeling group and hUC–MSCs treated alone group, while increased in the combination therapy group. Finally, the expression of 185 genes matched trend 1 and 148 genes matched trend 2 (Figure 4A). Following GO analysis showed that most of these genes were involved in cell proliferation and extracellular substrate deposition (Figure 4B). Deeply analysis showed that the expression of the genes which were positively related with cell proliferation, such as insulin receptor substrate 2 (Irs2) and Fos-related antigen 2 (Fosl2), were higher in the lungs of modeling group and hUC–MSCs treated alone group rather than that of normal control group and combination therapy grou p. While the genes which had negative relationship with cell proliferation expressed oppositely, such as peroxisome proliferator activated receptor gamma 2 (Pparg). The expression of the genes related with extracellular substrate deposition which could reflect the existence of myofibroblasts, such as collagen alpha-1(XXVIII) chain (Col28a1), matrix metallopeptidase 10 (*Mmp10*), Fgf2, and fibroblast growth factor 23 (Fgf23) were decreased in the therapy group than that in the modeling and hUC–MSCs treated alone group. In addition to this, the genes expressed by AECII cells, such as pulmonary surfactant-associated protein A (Sftpa1), pulmonary surfactant-associated protein D (Sftpd), pulmonary surfactant-associated protein C (Sftpc) and pulmonary surfactant-associated protein B (Sftpb), expressed higher in the combination therapy group than that in the modeling group and hUC–MSCs treated alone group (Figure 4C,D). While the expression of Pdgfrb and the downstream effectors of Pdgfr and Fgfr were not picked by the GO analysis, they were lower in the lungs of mouse treated with hUC–MSC and low-dose nintedanib together than hUC–MSC alone (*Supporting Information 2*: Figure S1).

From the results above, we inferred that hUC–MSCs might promote the proliferation of not only AECII cells but also fibroblasts as well, which might be the reason why single hUC–MSCs treatment had no effect on PF mice. While we combine hUC–MSCs with low-dose nintedanib, the latter partly inhibited the proliferation of fibroblasts and finally showed out some therapeutic effects.

### 3.4. Nintedanib Counteract the Promotional Effects of hUC–MSCs on the Proliferation of Fibroblasts

**Figure 5:**
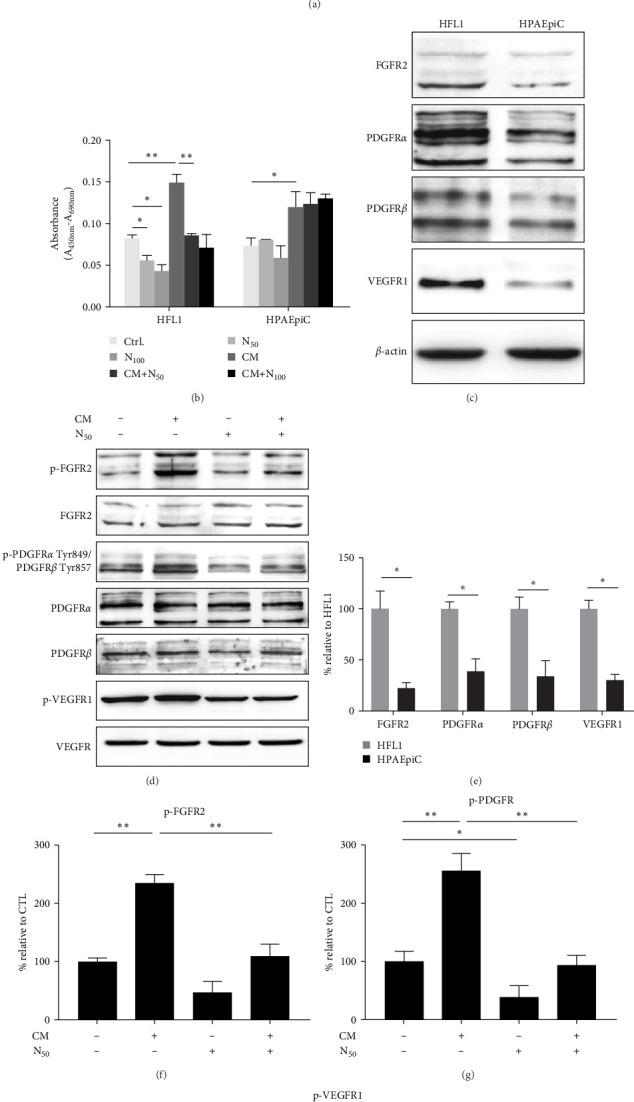
Nintedanib counteract the promotional effects of human umbilical cord–mesenchymal stem cells (hUC–MSCs) on the proliferation of fibroblasts. HFL1 and HPAEpiC were cultured with normal culture medium (Ctrl.), hUC–MSCs conditional medium (CM) with or with out nintedanib at different concentration (N_50_ represented 50 nM and N_100_ represented 100 nM). (A) Cell proliferation assay tested by cell counting kit-8 (CCK-8) at indicated times after culturing with different administration. (^a^*p* < 0.05 compared with Ctrl. ^b^*p* < 0.05 CM + N_50_ compared with CM. ^c^*p* < 0.05 CM + N_100_ compared with CM. ^d^*p* > 0.05 CM + N_100_ compared with CM + N_50_. ^e^*p* < 0.05 N_100_ compared with Ctrl.) (B) 5-bromo-2′-deoxyuridine (BrdU) incorporation assay. (C) Immunoblotting of the cell lysis. Quantitative evaluation is shown in (E). (D) Immunoblotting of the HFL1 cell lysis collected after culturing with indicated substrates for 72 h. Quantitative evaluation is shown in (F–H). Cell cultured in normal medium without hUC–MSCs CM and nintedanib is used as control group “CTL.” Shown is a representative of at least three independent experiments. For B, E–H, shown is mean ± SEM. *⁣*^*∗*^*p* < 0.05, *⁣*^*∗∗*^*p* < 0.01.

To confirm our hypothesis, we cultured the AECII line HPAEpiC and lung fibroblast cell line HFL1 in vivo. The CM of hUC–MSCs was used to treat both of the two cell lines with or without different concentration of nintedanib. Results had showed that CM of hUC–MSCs could promote both of the two cells proliferation, while nintedanib hindered the proliferation of HFL1 solely (Figure 5A,B).

Nintedanib is a tyrosine kinase inhibitor which can inhibit the activation of PDGFR, FGFR, and VEGFR. PDGF, FGF, and VEGF were included in the factors secreted by UC–MSCs. FGFR2, PDGFR*α*, PDGFR*β*, and VEGFR1 are highly expressed by HFL1, while minorly expressed by HPAEpiC (Figure 5C,E). The phosphorylation of FGFR2, PDGFR*α*/*β*, and VEGFR1 could be increased by the CM of UC–MSCs. However, nintedanib neutralized the promotional effects of CM on FGFR2, PDGFR*α*/*β*, and VEGFR1 phosphorylation in HFL1 (Figure 5D,F,G,H). In conclusion, nintedanib might impede the proliferation of fibroblasts induced by hUC–MSCs conditioned medium through inhibiting the activation of FGFR, PDGFR, and VEGFR, thus, playing a synergistic therapeutic role with hUC–MSCs on PF. Meanwhile, it had little influence on alveolar epithelial proliferation.

## 4. Discussion

MSCs can be cultured pretty easily and can be obtained from medical waste including umbilical cord tissue. Allogeneic hUC–MSC injection is safe as its low antigenicity. According to the data from ClinicalTrials.gov until November 1, 2022, hUC–MSCs have been evaluated in more than 200 in a variety of fields, with 24 clinical trials related to fibrosis diseases and three clinical trials related to lung fibrosis. There are many preclinical studies focusing on the preventive effects of hUC–MSCs on PF. However, few researchers costed attention to the therapeutic roles of hUC–MSCs on lung fibrosis. This is related to previous studies suggesting that the administration of MSCs after fibrosis formation may aggravate PF and lead to further deterioration of lung function. We also observed that intervention with hUC–MSCs alone did not reverse PF at 7 days after bleomycin modeling and hUC–MSCs administration without bleomycin seemed to bring some positive Masson's trichrome staining reaction (Figure 1). As fibrosis is one of the major adverse events after MSCs infusion [22] that caution should be taken when using MSCs to treat fibrosis diseases.

Lung injury and subsequent fibrosis is a complex process and overlapping continuous process that can be regulated by the presence of various types of cells, growth factors, cytokines, and extracellular matrix elements. The therapeutic effects of hUC–MSCs primarily rely on a paracrine function. First, hUC–MSCs have immunomodulatory property which is determined by the secretion of anti-inflammatory factors such as PGE2, TGF*β*, IDO, and nitric oxide. Second, they promote angiogenesis and improve vascular circulation through the secretion of angiogenetic factors, such as VEGF. Finally, through producing variable trophic factors such as FGF2, EGF, KGF, HGF, GM-CSF, IGF, and PDGF, they could promote the survival of neighboring stem and progenitor cells [23, 24]. Among the growth factors secreted by hUC–MSCs, KGF, HGF, EGF, and GM-CSF promotes ATII cell proliferation [25], while VEGF, PDGF, and FGF2 stimulates fibroblast proliferation [26, 27]. In this research, we also observed that the CM of hUC–MSCs promotes proliferation of AECIIs and lung fibroblasts in parallel. This may be the reason why hUC–MSCs administration alone was ineffective and even aggravates PF after 7 days of bleomycin modeling. While there was no significant difference of myofibroblasts proliferation in the lungs between the mouse treated with hUC–MSCs and those without hUC–MSCs treatment after bleomycin modeling. This may be related to the fact that the proliferation of myofibroblasts in the lungs of the mice in the model group has reached a ceiling effect.

To counteract the detrimental effect of hUC–MSCs, retaining the stimulative effect on ATII cell proliferation, we used nintedanib as a combination with hUC–MSCs. Nintedanib is an oral tyrosine kinase inhibitor, also known as a triple angiokinase inhibitor, inhibiting three major kinases involved in angiogenesis, including PDGFRs、FGFRs, and VEGFRs [28]. Nintedanib was shown to inhibit proliferation of normal human lung fibroblasts in vitro and to inhibit PDGF-BB, FGF2, and VEGF-induced proliferation of human lung fibroblasts from patients with IPF and control donors [26], while had no effect on the proliferation of human epithelial cell lines [28]. In this research, we not only confirmed the directly impeding effect of nintedanib, but also observed that nintedanib reversed the facilitation of hUC–MSCs CM on lung fibroblast proliferation which might be related to the antagonism of nintedanib on FGFR, PDGFR, and VEGFR activation. However, the auxo-action of hUC–MSCs CM on lung epithelial cells proliferation was not disturbed, even the minute expression of FGFR, PDGFR, and VEGFR on the lung epithelial cell surface. KGF1 (also called as FGF7) and KGF2 (also called as FGF10) were reported as a growth factor of ATII cells which increase the epithelial survival after injury through FGFR2b signaling [29–31]. But the proliferation of ATII cells were not inhibited by nintedanib, which indicated that other signaling pathways involved in ATII cells proliferation maintenance except for FGFR2 signaling.

Except of the genes included in FGFR, PDGFR, and VEGFR signaling pathways and related to the proliferation of ATII cells and fibroblasts, we still observed other proliferation related genes (such as Irs2 and Fosl2) which decreased with nintedanib administration. As Irs2 had been reported to mediate the stimulation of insulin on fibroblast proliferation [32], nintedanib might impede fibroblast proliferation through Irs2 reduction. In addition, Fosl2 has been found to promote fibrosis through fibroblast activation [33] and promote VEGF-independent angiogenesis by transcriptionally activating Wnt5a in fibroblasts [34]. Although the effect of nintedanib on fosl2 has remained elusive, the synergistic therapeutic effect of nintedanib and hUC–MSCs on PF probably related to fosl2 decrease.

As MSCs serve as important regulators of tissue homeostasis and regeneration, it is very promising to try to apply it to the treatment of chronic “irreversible” diseases. In this research, we use nintedanib to eliminate the pro-proliferation effects of hUC–MSC on lung fibroblasts, retaining the promoting effect of hUC–MSC cells on ATII cell proliferation. But there may be a more perfect way to improve the repair effect of hUC–MSC on PF. There is a growing interest in the potential of lung-resident MSCs (LR-MSCs) to help identify more targeted treatments for respiratory pathologies [35]. LR-MSCs are an integral part of the lung injury repair process [36–39]. However, some research showed that LR-MSCs can differentiate to myofibroblast, which contribute to ECM deposition and disease progression in IPF as well [40–44]. Comparative studies on exogenous MSCs (such as UC-MSCs) and LR-MSCs will help to better utilize the beneficial aspects of exogenous MSCs for PF and promote the therapeutic application of exogenous MSCs for PF related diseases. Human embryonic stem cells (hESCs) or human induced pluripotent stem cells (hiPSCs) derived lung tissue-specific functional cells (such as ATII cells) may also be a candidate for the treatment of PF [45, 46]. But the clinical application of these two types of cells has been limited by the ethical criticism for hESCs, unstable genome of hiPSCs [47], immunological rejection [48], and the potential for tumor formation [49]. hUC–MSCs derived extracellular vesicles (EVs, including exosomes and microvesicles) may be another choice for the treatment of PF. Without the implantation of cells themselves, the use of exosomes can prevent the implanted cells from transforming into fibroblasts. Although EVs have shown therapeutic benefits in many preclinical models of lung injury and disease [50–52], they may also contain two types of factors that promote the growth of ATII cells and fibroblasts simultaneously. On the other hand, the application of MSCs derived EVs are also obstructed by cumbersome isolation techniques and their instability.

As mentioned in our article, hUC–MSCs may promote ATII proliferation through a paracrine mechanism, thereby, exerting a synergistic therapeutic effect with nintedanib on lung fibrosis. To avoid the side effects of cell infusion, subsequent studies can focus on the set of factors secreted by hUC–MSCs that promote the proliferation of ATII cells. In addition to the more studied growth factors (e.g., HGF and EGF), some special factors which may be related to ATII cell proliferation should also be considered for inclusion in this factor set. Such as PPAR*γ*, the expression of which was higher in the lung tissue after hUC–MSCs interfered with PF in our research. The effects of PPAR*γ* activation on the proliferation of epithelial cells are different between the tissues [53, 54]. For lung tissue, PPAR*γ* is necessary for normal lung maturation [55]. Combined with our observation, PPAR*γ* activation may have a positive effect on the proliferation of ATII cells. Therefore, the PPAR*γ* agonists (e.g., pioglitazone and rosiglitazone) could be considered as one of the combinational chemicals with nintadanib for the treatment of PF.

## 5. Conclusion

Looking forward new treatments for patients with IPF is very challenging. We provide preclinical research to support for the combinational therapy of nintedanib and hUC–MSCs on PF. The combined application can eliminate the facilitation of hUC–MSCs on fibroblasts proliferation, maintaining their mitogenic activity on ATII cells. This is expected to provide an option for subsequent clinical treatment research of PF.

## Data Availability

All data generated and analyzed during this study are included in this published article. Further inquiries can be directed to the corresponding authors.

## Supporting Information

Supporting Information 1 Table S1: primers used in this research.

Supporting Information 2 Figure S1: expression of the downstream effectors of Pdgfr and Fgf4 were suppressed by low-dose nintedanib in the lung tissues after bleomycin modeling. RNA-Seq had been applied for the differentially expressed genes detection in lung tissues from indicated mouse groups. “Ctrl.”, saline injected intratracheally (i.t.) alone. For model control group “B”, 3.0 mg/kg bleomycin had been i.t. For group treated by hUC–MSCs alone “BM”, 2 × 10^5^ hUC–MSCs in 80 μl saline had been injected intravenously (i.v.) at 7 days after modeling. For combination treatment group “BMN10”, 2 × 10^5^ hUC–MSCs in 80 μl saline had been i.v. at 7 days after modeling and 10 mg/kg nintedanib had been delivered intragastically (i.g.) daily from the 7 to 20 days after modeling. For the group treated by low-dose nintedanib alone “BN10,” 10 mg/kg nintedanib had been delivered i.g. daily from the 7 to 20 days after modeling without hUC–MSCs. *n* = 3 mice each group.
