# Supplementary material for: Human Umbilical Cord–Mesenchymal Stem Cells Combined With Low Dosage Nintedanib Rather Than Using Alone Mitigates Pulmonary Fibrosis in Mice
Source: Stem Cells Int. 2025 Jan 7;2025:9445735. doi: 10.1155/sci/9445735 (PMC11732289; doi:10.1155/sci/9445735)
Supplement: Supporting Information 1 — Table S1: primers used in this research. [file 9445735.f1.pdf]

Supplementary Table 1

Supplementary Table 1. Primers used in this research.

genes	Primers（5‘->3’）	
**Irs2**	Forward	CCAGTAAACGGAGGTGGCTACA
	Reverse	CCATAGACAGCTTGGAGCCACA
**Fosl2**	Forward	AGGAGGAGAAGCGTCGAATCCG
	Reverse	CCAGACTTCTCCTCTTCCAGCT
**Pparg**	Forward	GTACTGTCGGTTTCAGAAGTGCC
	Reverse	ATCTCCGCCAACAGCTTCTCCT
**Col28a1**	Forward	GCCTGGTCTTAAAGGAGAACCTG
	Reverse	AGCCATCACCTTTGAGTCCTGG
**Mmp10**	Forward	TGCTGCCTATGAGGCTCACAAC
	Reverse	GGAGGAAAACCGAGAGTGTGGA
**Fgf2**	Forward	AAGCGGCTCTACTGCAAGAACG
	Reverse	CCTTGATAGACACAACTCCTCTC
**Fgf23**	Forward	AACAGGAGCCATGACTCGAAGG
	Reverse	CGTCATAGCCATTCTCCAGCGT
**Sftpa1**	Forward	ACCTGGATGAGGAGCTTCAGAC
	Reverse	CTGACTGCCCATTGGTGGAAAAG
**Sftpd**	Forward	AGGTCCAGTTGGACCCAAAGGA
	Reverse	CTGGTTTGCCTTGAGGTCCTATG
**Sftpc**	Forward	GTCCTCGTTGTCGTGGTGATTG
	Reverse	AAGGTAGCGATGGTGTCTGCTC
**Sftpb**	Forward	TGTCCTCCGATGTTCCACTGAG
	Reverse	AGCCTGTTCACTGGTGTTCCAG
