# Supplementary figures and images for: Human Umbilical Cord–Mesenchymal Stem Cells Combined With Low Dosage Nintedanib Rather Than Using Alone Mitigates Pulmonary Fibrosis in Mice
Source: Stem Cells Int. 2025 Jan 7;2025:9445735. doi: 10.1155/sci/9445735 (PMC11732289; doi:10.1155/sci/9445735)

## Supplementary Fig. 1

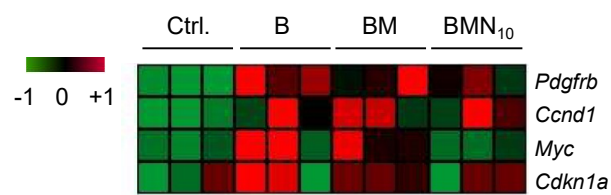

Supplement: Supporting Information 2 — Figure S1: expression of the downstream effectors of Pdgfr and Fgf4 were suppressed by low-dose nintedanib in the lung tissues after bleomycin modeling. RNA-Seq had been applied for the differentially expressed genes detection in lung tissues from indicated mouse groups. “Ctrl.”, saline injected intratracheally (i.t.) alone. For model control group “B”, 3.0 mg/kg bleomycin had been i.t. For group treated by hUC–MSCs alone “BM”, 2 × 10^5^ hUC–MSCs in 80 μl saline had been injected intravenously (i.v.) at 7 days after modeling. For combination treatment group “BMN10”, 2 × 10^5^ hUC–MSCs in 80 μl saline had been i.v. at 7 days after modeling and 10 mg/kg nintedanib had been delivered intragastically (i.g.) daily from the 7 to 20 days after modeling. For the group treated by low-dose nintedanib alone “BN10,” 10 mg/kg nintedanib had been delivered i.g. daily from the 7 to 20 days after modeling without hUC–MSCs. n = 3 mice each group. [file 9445735.f2.pdf]
